# Effect of Strain Rate on the Mechanical Properties of Cu/Ni Clad Foils

**DOI:** 10.3390/ma14226846

**Published:** 2021-11-12

**Authors:** Haiyang Wang, Chuanjie Wang, Linfu Zhang, Gang Chen, Qiang Zhu, Peng Zhang

**Affiliations:** 1 Key Laboratory of Micro-Systems and Micro-Structures Manufacturing, Harbin Institute of Technology, Ministry of Education, Harbin 150080, China; 18s030154@stu.hit.edu.cn; 2 School of Materials Science and Engineering, Harbin Institute of Technology at Weihai, Weihai 264209, China; 17863130516@163.com (L.Z.); cg@hitwh.edu.cn (G.C.); zhuqiang@hit.edu.cn (Q.Z.)

**Keywords:** Cu/Ni clad foil, strain rate sensitivity, microforming

## Abstract

The performance of clad foils in microforming deserves to be studied extensively, where the strain rate sensitivity of the clad foil concerning the forming performance is a crucial factor. In this paper, the strain rate sensitivity of the mechanical properties of coarse-grained (CG) Cu/Ni clad foils in the quasi-static strain rate range ($\dot{\varepsilon }=10^{-4}\;s^{-1}\sim 10^{-1\;}s^{-1}$) is explored by uniaxial tensile tests under different strain rates. The results show that the strength and ductility increase with strain rate, and the strain rate sensitivity *m* value is in the range of 0.012~0.015, which is three times the value of m for CG pure Cu. The fracture morphology shows that slip bands with different directions are entangled in localized areas near the interface layer. Molecular dynamics simulations demonstrate the formation of many edged dislocations at the Cu/Ni clad foils interface due to a mismatch interface. The improved ductility and strain rate sensitivity is attributed to the interaction and plugging of the edged dislocations with high density in the interface layer. Additionally, the influence of size effect on mechanical properties is consistently present in the quasi-static strain rate range. This paper helps to understand the strain rate sensitivity of CG clad foils and to develop clad foils in microforming processes.

## 1. Introduction

Micro-components are widely available in various advanced industries, including aerospace, electronics, energy batteries, etc. Microforming technology stands out among microfabrication technologies as the primary approach to manufacturing micro-components due to its high throughput, precision, and efficiency [1,2]. However, the entire miniaturization of the processing system introduces many challenges in microforming [3,4,5]. One of the most prominent contradictions is the significant reduction in material ductility caused by size effects [3,6]. The size effect is the notable decrease in strength and ductility of thin sheets when the grain size and thickness are reduced below a critical value [7,8]. It presents potential hazards for the material formability and reliability of micro components [9]. Thus, various attempts must be employed to inhibit premature fracture, including enhancing material properties and optimizing process parameters. Among them, increasing the strain rate becomes an excellent choice for materials with high strain rate sensitivity.

Numerous studies have investigated the effect of size effects on material ductility [10,11,12,13]. Fu et al. [14] conducted tensile tests on pure Cu foils with different thicknesses and grain sizes to investigate the size effect on fracture behavior. They found that the fracture stress and strain decreased as the thickness to grain size ratio (*t*/*d*) decreased. The fracture strain decreased significantly as *t*/*d* < 2. Yang et al. [15] found that the elongation of pure Cu sheets decreased significantly when the specimen thickness was below the critical thickness. Tang et al. [16] suggested a transition value for *t*/*d* where the fracture strain significantly dropped when *t*/*d* was below this value. Furushima et al. [17] observed that the fracture strain of pure Cu sheets decreased as the ratio of surface roughness to thickness increased. The reasons for the sudden decrease in fracture strain are manifold. They include the greater contribution of the properties of individual grains, the amplification of surface defects, and the reduction in the hardening capacity due to less activation of the slip system [14]. The most direct manifestation is the change of fracture mode from a normal fracture to a single-crystal slip mode. Thus, various approaches must be employed to inhibit premature fracture, including enhancement of material properties and optimizing process parameters. Among them, increasing the strain rate becomes an excellent choice for materials with high strain rate sensitivity [18].

However, the strain rate sensitivity is low for coarse-grained FCC metals such as Cu and Ni [19,20] ($m_{\mathrm{Cu}},m_{\mathrm{Ni}}=\sim 0.004$). Moreover, the elongation of coarse-grained (CG) Cu and Ni decreases slightly with strain rate. This phenomenon means that strain rate does not play a role for CG Cu or Ni. Thus, finding a material with high strain rate sensitivity is a necessary task. The clad foils seem to be an ideal material. Several studies have found that clad foils with ultrafine-grained (UFG) substrate layers exhibit higher strain rate sensitivity. Tan et al. [21] found that the UFG Cu/Ni multilayer foils prepared by electrodeposition exhibited high strain rate sensitivity. Strength and elongation increased with increasing strain rate from $\dot{\varepsilon }=1\times 10^{-5}\;s^{-1}$ to $\dot{\varepsilon }=1\times 10^{-2}\;s^{-1}$. They concluded that the stress gradient near the interface layer increased with increasing strain rate due to the higher strain rate sensitivity of the UFG Ni layer than the Cu layer. It promoted dislocation accumulation and inhibited dislocation recovery to increase strength and ductility. Zheng et al. [22] found the same phenomenon via quasi-static uniaxial tensile experiments on UFG Cu/Ni multilayer foils. Under high strain conditions, the strain rate sensitivity *m* value reached 0.017, which is four times as high as that of UFG Cu and Ni. They demonstrated that Cu/Ni multilayer foils exhibited larger back stress at high strain rate conditions by loading-unloading-loading experiments. High back stress enhanced material strength and promoted work hardening, which helped to achieve larger elongation. However, due to process defects, the UFG Cu/Ni multilayer foils prepared by electrodeposition cannot be mass-produced. The CG sheets and clad foils prepared by rolling and heat treatment are widely used materials. Since CG Cu and Ni exhibit very low strain rate sensitivity [19], the stress gradient at the interface layer remains stable with increasing strain rates. The model proposed by Tan and Fu et al. [21,22] cannot be applied to CG Cu/Ni clad foils. Thus, whether CG Cu/Ni clad foils exhibit high strain rate sensitivity needs to be investigated experimentally.

In this paper, Cu/Ni clad foils with 100 μm thickness prepared by cold rolling were annealed to obtain a CG Cu and Ni layer. The strain rate sensitivity of the Cu/Ni clad foil was tested by uniaxial tensile tests with different strain rates. The reason for the variation of ductility with strain rate was investigated by fracture observation. The study of strain rate sensitivity of Cu/Ni clad foils at the mesoscale is carried out to confirm the strengthening effect of the interface on the *m* value and to investigate the performance of the clad foil at different strain rates. This paper will guide the implementation of clad foils in microforming processes.

## 2. Materials and Methods

**Figure 1:**
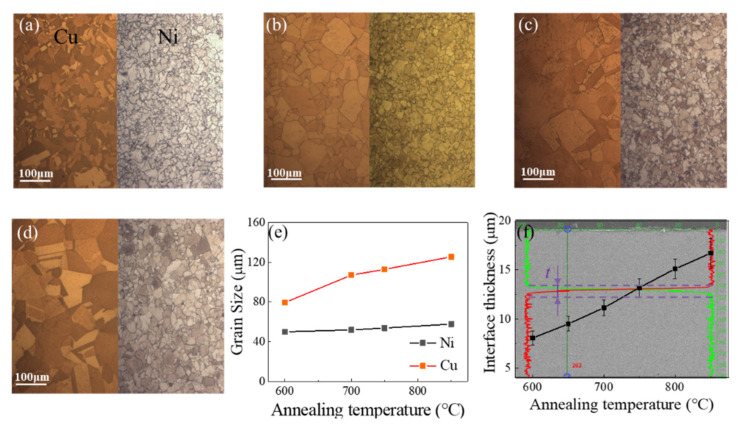
Microstructures of Cu and Ni layer under different annealing temperatures: (**a**) 600 °C, (**b**) 700 °C, (**c**) 750 °C, (**d**) 850 °C, (**e**) variation of grain size of Cu and Ni layers after annealing, and (**f**) variation of thicknesses of the interface layer.

**Figure 2:**
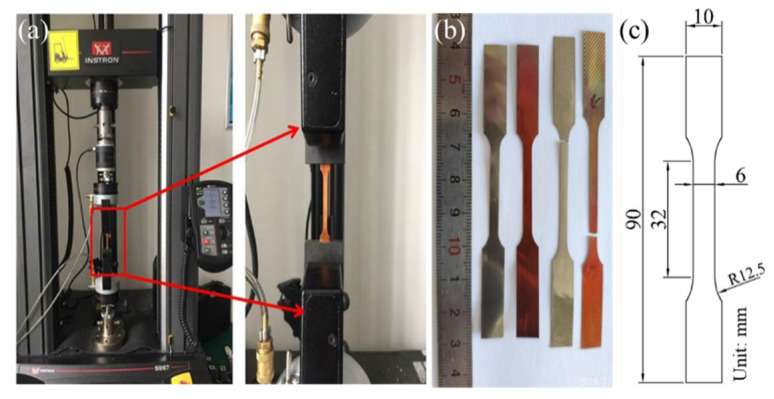
(**a**) Universal material testing machine; (**b**) tensile specimens; (**c**) geometric dimensions of tensile specimens.

The Cu/Ni clad foils (Huihua Composite Materials Co., Ltd., Yixing, China), prepared by cold rolling with a thickness of 100 μm were studied by uniaxial tensile tests at different strain rates. The initial thickness ratio of Cu to Ni was 5.5:4.5. The annealing temperatures and dwelling time were 600 °C for 1 h, 700 °C for 1 h, 750 °C for 1 h, and 850 °C for 1 h, respectively, before being cooled in the air at room temperature to obtain various microstructures. The microstructure on the surface was observed by Optical Microscope (OM) (Olympus Corporation, Tokyo, Japan), as shown in Figure 1a–d. The grain size at different heat treatment conditions was shown in Figure 1e. The thickness of the interface layer was measured by Energy Dispersive X-Ray (EDX) (Zeiss, Oberkochen, Germany), as shown in Figure 1f. The grain size of the Cu and Ni layer and the thickness of the interface layer increased with increasing annealing temperature in Figure 1e,f. The uniaxial tensile tests were performed on an INSTRON 5967 testing machine (Instron, Boston, MA, USA) with strain rates ranging from $\dot{\varepsilon }=1\times 10^{-4}\;s^{-1}$ to $\dot{\varepsilon }=1\times 10^{-1}\;s^{-1}$, as shown in Figure 2a. The geometrical dimensions of the specimen are in Figure 2b. At least 3–5 specimens were tested under these specific experimental conditions. The fracture morphology of Cu/Ni clad foils was observed using a Scanning Electron Microscope (SEM) (Zeiss, Oberkochen, Germany).

Molecular Dynamics (MD) simulations were performed to investigate the interfacial structure and atomic diffusion behavior during annealing. The MD simulations were performed using the Large-scale Atomic/Molecular Massively Parallel Simulator (LAMMPS-22 August 2018) [17]. The Open Visualization Tool (OVITO-BASIC 3.1.0) [18] with common neighbor analysis (CNA) and dislocation analysis (DXA) method was employed to analyze the microstructure of models from MD. In the simulation models, the interactions between atoms (Cu-Cu, Ni-Ni and Cu-Ni) were described by the EAM potential constructed by Fischer et al. [23]. The Cu/Ni clad foil model with the semi-coherent interface (SCI) was constructed by joining Cu and Ni crystals of the same crystal orientations and coordinates: the *x*-axis along [111] the *y*-axis along $\left[11\bar{2}\right]$ the *z*-axis $\left[\bar{1}10\right]$. Periodic boundary conditions were applied for three dimensions. To minimize internal stresses in the model, the number of lattices (N) along the *y* and *z*-axis should satisfy $N\times a_{\mathrm{Cu}}=\left(N+1\right)\times a_{\mathrm{Ni}}$. Due to $a_{\mathrm{Cu}}=0.3615$ and $a_{\mathrm{Ni}}=0.3520$, it was obtained that N≈37. Then, the simulation cell dimensions were ~32.7 nm and ~18.9 nm along the *y* and *z* directions. All the MD simulations were performed using the Verlet integration algorithm with a time step of 1.0 fs. The models were minimized energy, using a conjugate gradient algorithm. Then, the optimized Cu/Ni clad foil models were annealed to 600 °C, 700 °C, and 850 °C by a rate of 1.5 K/ps in an isothermal-isobaric. Then, the system quickly dropped to 25 °C. The clad structures were equilibrated to 50 ps in NPT ensemble for obtaining a stable equilibrium system.

## 3. Results

**Figure 3:**
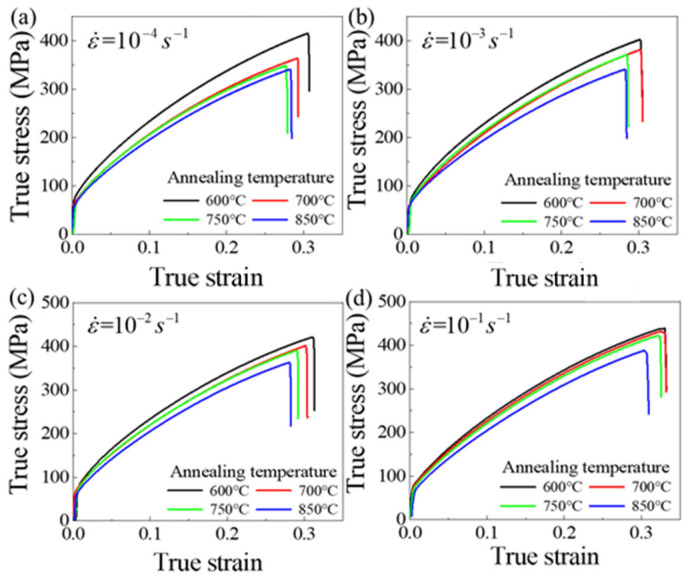
True stress–strain curves of specimens with various annealing conditions at: (**a**) $\dot{\varepsilon }=10^{-4}{\;s}^{-1}$, (**b**) $\dot{\varepsilon }=10^{-3}\;s^{-1}$, (**c**) $\dot{\varepsilon }=10^{-2}\;s^{-1}$, and (**d**) $\dot{\varepsilon }=10^{-1}\;s^{-1}$.

**Figure 4:**
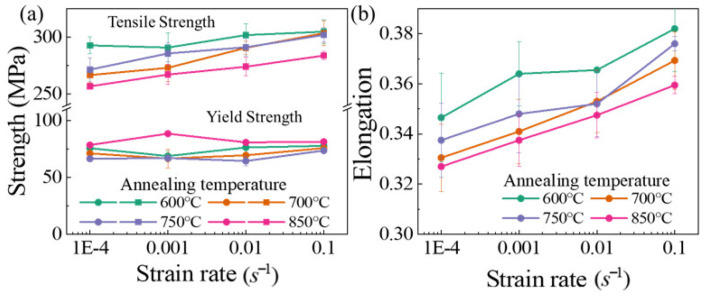
Mechanical properties of specimens at various strain rates: (**a**) strength, (**b**) elongation.

**Figure 5:**
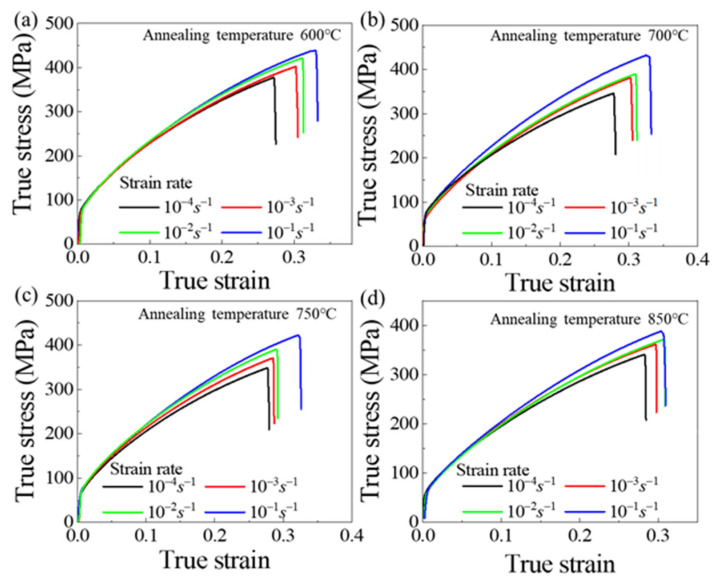
True stress–strain curves of specimens with different annealing conditions at various strain rates: (**a**) 600 °C, (**b**) 700 °C, (**c**) 750 °C, and (**d**) 850 °C.

**Figure 6:**
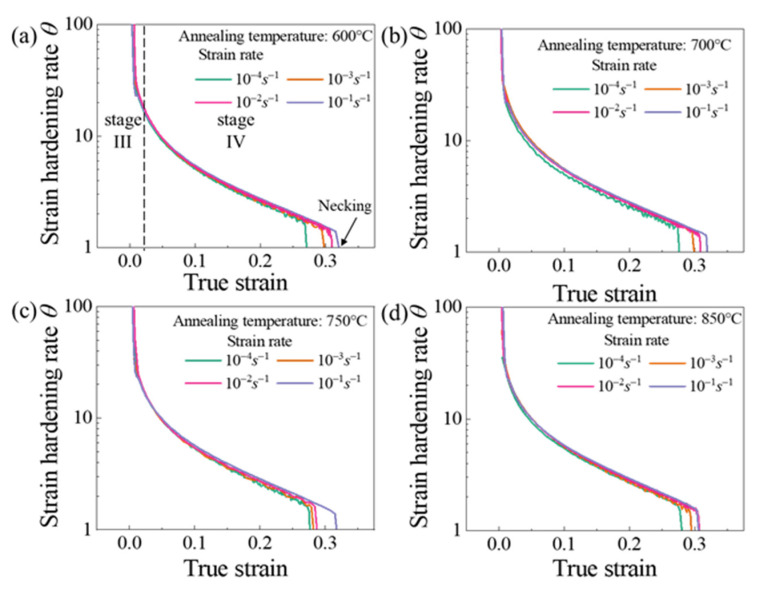
Normalized strain hardening rate (*θ*) vs. true strain of specimens with different annealing conditions at various strain rates: (**a**) 600 °C, (**b**) 700 °C, (**c**) 750 °C, and (**d**) 850 °C.

Figure 3 shows the influence of annealing temperature on the flow stress of Cu/Ni clad foils. The flow stress continuously decreased under various strain rates. This means that size effects consistently affect the mechanical properties of Cu/Ni clad foils over a quasi-static strain rate range. Figure 4a presented the variation of yield and tensile strengths for specimens with different grain sizes at $\dot{\varepsilon }=1\times 10^{-4}\;s^{-1}$ to $\dot{\varepsilon }=1\times 10^{-1}\;s^{-1}$. Yield strength remained almost unchanged, and tensile strength increased slightly with increasing $\dot{\varepsilon }$. Figure 4b showed that the elongation of Cu/Ni clad foils increased with $\dot{\varepsilon }$. Strain rate performed a similar effect on the ductility enhancement of specimens for various annealing conditions. The elongation increased by ~10% when the strain rate rose from $10^{-4}\;s^{-1}$ to $10^{-1}\;s^{-1}$. Additionally, the elongation decreased slightly with the increase of annealing temperature. Figure 5 illustrated the true stress–strain curves of specimens with different annealing temperatures at different $\dot{\varepsilon }$. The true stress and fracture strain increased significantly. On this basis, the strain hardening rate *θ* (θ=(∂σ/∂ε)/σ) was calculated based on the data in Figure 5. When *θ* = 1, the strain hardening index was equal to the true stress ∂σ/∂ε=σ; the necking is determined as the starting point [24]. Figure 6 illustrated that strain hardening rate, *θ*, displayed an insignificant increase as the $\dot{\varepsilon }$ increased. Thus, the increase in strain rate did not provide an appreciable improvement to the elongation. The strain hardening rate curves showed that the first stage of work hardening (easy slip stage) and the second stage (linear hardening stage) vanished, due to the lower yield strength. The strain hardening rate curves consisted mainly of the third (III) stage (dynamic recovery stage), and the fourth (IV) stage (strain hardening stage), where the third stage was completed within a small strain and the fourth stage occupied a considerable proportion. As the strain rate increased, the length of the fourth stage increased to inhibit necking.

**Figure 7:**
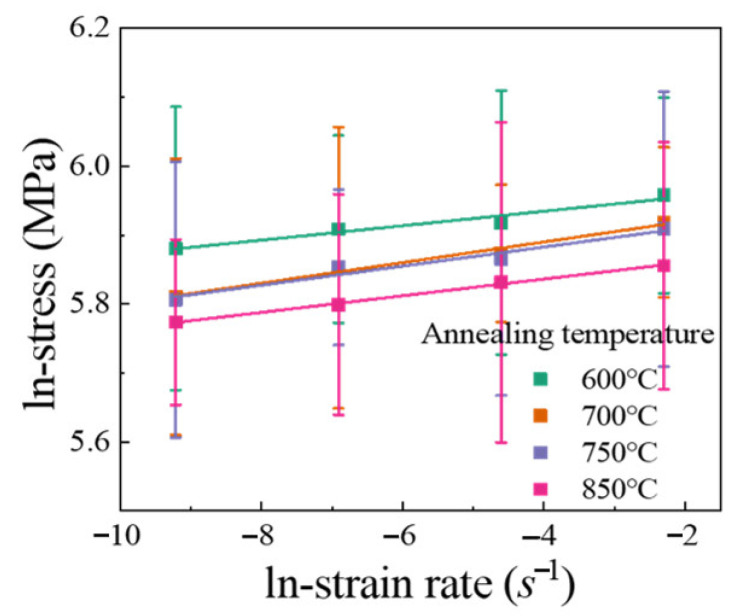
ln-stress vs. ln-strain rate curves of specimens with different annealing conditions.

**Figure 8:**
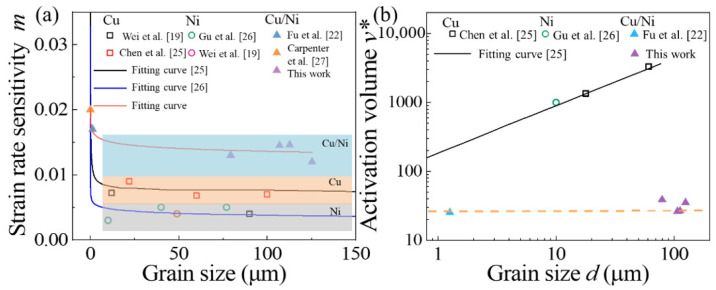
(**a**)Variation of *m*; (**b**) variation of v* as a function of *d* for Cu, Ni, and Cu/Ni clad foils using experimental data from prior papers and the present work.

Figure 7 shows the change curves between in-stress and in-strain rate. The corresponding strain rate sensitivity index *m* value was calculated as the slope of these lines. The *m* value exhibited independence of grain size. Figure 8a presented the variation of *m* values as a function of grain size *d* for Cu, Ni, and Cu/Ni clad foils, including literature data from various test methods [19,22,25,26,27]. The trends of the three fitted curves corresponding to Cu, Ni, and Cu/Ni clad foils were consistent. The *m* values slightly increased as *d* decreased from macroscale to submicron scales. Additionally, the *m* value significantly increased when *d* decreased below 1000 nm. Alternatively, the *m* value of Cu/Ni clad foil was significantly higher than that of pure Cu and Ni for the CG materials. It had been demonstrated that the increasing stress gradient at the interface layer with strain rate contributed to both the strength and ductility of the UFG/NC clad foil due to the larger *m* value of NC Cu and Ni. For CG Cu/Ni clad foils, this increase in *m* value was strongly linked to strain rate sensitivity in the plastic deformation of the interface layer. Figure 8b showed the activation volume v* versus grain size. The activation volume of CG Cu/Ni clad foils was considerably lower than that of CG Cu and Ni. For pure Cu and Ni, the activation volume linearly decreased as the grain size decreased. However, the activation volume was independent of the grain size for Cu/Ni clad foils.

**Figure 9:**
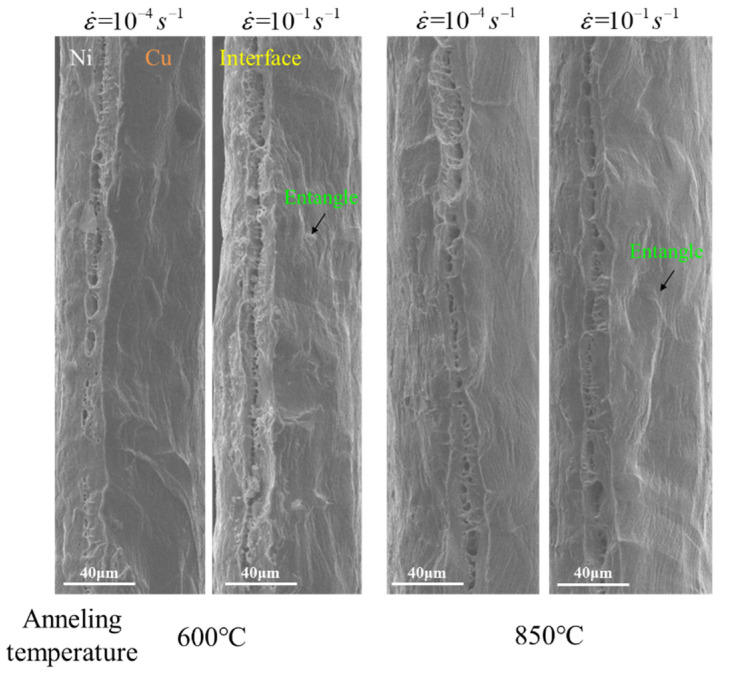
Fracture topography of specimens with different annealing conditions at various strain rates.

Figure 9 displayed the fracture morphology of the specimens under various annealing conditions at different strain rates. As $\dot{\varepsilon }=1\times 10^{-4}\;s^{-1}$, the fracture surface exhibited smooth and a few directionally consistent slip marks. When the $\dot{\varepsilon }$ increased to $1\times 10^{-1}{\;s}^{-1}$, the fracture surface became rough and slip marks in different directions appeared in localized areas near the interface layer and became entangled, related to the obstructive effect of forest dislocations.

**Figure 10:**
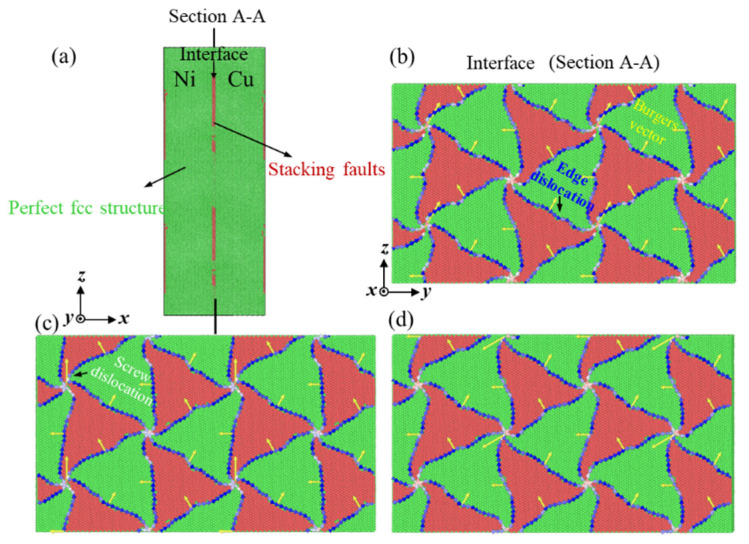
Configurations of Cu/Ni clad foil with semi-coherent: (**a**) CNA analysis results, DXA analysis results, (**b**) annealed at 600 °C, (**c**) annealed at 700 °C, (**d**) annealed 850 °C.

The interfacial behavior of Cu/Ni clad foils was investigated by molecular dynamics simulations. The interface of Cu/Ni clad foil was treated by annealing and air-cooling of the relaxation process. Figure 10a showed the configurations of Cu/Ni clad structures with semi-coherent. After energy minimization, stacking fault (SF) existed at the interface in Figure 10b. Figure 10b–d presented the relaxed semi-coherent interface of models with various annealing conditions. This can be divided into four areas. In the relaxation process, six kinds of Shockley partial dislocations, $\pm b_{1}$, $\pm b_{2}$, $\pm b_{3}$, ($b_{1}=a\left[11\bar{2}\right]/6$, $b_{2}=a\left[1\bar{2}1\right]/6$, $b_{3}=a\left[\bar{2}11\right]/6$), for between FCC and SF areas, and cross the nodes [28]. The yellow arrow in Figure 10b–d was the Burgers vector. The direction of the Burgers vector was approximately perpendicular to the direction of most dislocation lines. This means that there were many edged Shockley partial dislocations at the interface. With the increase of heat treatment temperature, the dislocations at the interface still existed, and the dislocation lines were slightly distorted. Additionally, a few screw dislocations were formed at the nodes.

## 4. Discussion

Overall, the strain rate has little effect on the strength of pure Cu and Ni sheets in tensile tests at room temperature. Compared with single-layer metal sheets, the Cu/Ni clad foils have a unique interface layer [29]. The interface layer dramatically influences the mechanical properties, plastic deformation, and fracture behavior of clad foils. Yield strength of the clad foils is influenced by the properties and thickness of the interface layer [29] and nanoscale interfacial layers greatly improve the strength of compound foils by limiting dislocation motion [30]. The strain gradient induced by the Cu/Ni interface may introduce a high density of GND, which retards the plastic instability along the thickness direction, and better interfacial adhesion facilitates the co-deformation ability of adjacent components to achieve co-necking [31]. The stress field and dislocation sources near the interface layer may be fundamental to obtain good strength-ductility coordination of the clad foils [32,33]. Many investigations on the strain rate of compound foils have been implemented to show that the density of mismatch dislocations at the interface affects the strain rate sensitivity due to the interaction of dislocation motion and mismatch [27,34]. The following discussion includes the enhancement mechanism of interfacial dislocations for strain rate sensitivity and ductility.

The results of molecular dynamics simulations in Figure 10 reveal that a lot of edged dislocations and a few screw dislocations are present at the interface layer. The nodes and initial dislocations are used as dislocation sources to form new dislocations with strain continuity. Furthermore, the dislocations not only develop in the plane of the interface layer but also extend to the Cu and Ni layers [25]. The situation becomes more complicated for the interface layer in the experiment. This condition creates an interface layer with high dislocation density. It facilitates the strength of the clad foils.

From the perspective of strain rate sensitivity, the *m* value of CG Cu/Ni clad foils is obviously higher than that of CG Cu and Ni, which is attributed to the heterogeneous interface of clad foils. For CG Cu and Ni metals, the main obstacle to dislocation motion is caused by forest dislocations, leading to rate dependence of flow stress through thermal activation [35]. Becker et al. [19] argued that the thermal activation volume $v^{*}$ was calculated as:

(1) $$v^{*}=b\times \xi \times l^{*}$$

where *b* is the Burgers vector of the dislocations, *ξ* is the distance swept out by the mobile dislocation during one activation, and $l^{*}$ is the length of the dislocation segment involved in the thermal activation (or the Friedel sampling length that scales with the average contact distance between two obstacles). For CG fcc metals, $l^{*}$ is the average forest spacing. Based on the physically based strain rate sensitivity *S*, its engineering strain rate sensitivity index *m* value and activation volume $v^{*}$ can be written as:

(2) $$m=\frac{S}{\tau }=\frac{k_{B}T}{\tau v^{*}}=\frac{k_{B}T}{\tau \times b\times \xi \times l^{*}}$$

where *T* is the absolute temperature, and *k_B_* is the Boltzmann constant.

For Cu/Ni clad foils, many edged dislocations and a few screw dislocations are created at the interface after annealing in Figure 10c, which effectively reduces the $l^{*}$ in Figure 8b [19]. Additionally, as the activation volume decreases, this leads to an increase in *m* value.

From the perspective of ductility, the ductility of Cu/Ni clad foils increases with $\dot{\varepsilon }$. As the strain rate increases, the activated mobile dislocations at the interface are greatly increased. Many dislocations undergo interaction, and dislocations are constantly nucleating [28]. It leads to larger strain hardening to improve strength. Expectedly, the increased strain rate also enhances the ductility of the CG Cu/Ni clad foils. Tan et al. [21] suggested that the stress gradient near the interface layer was the primary reason for strengthening the strength and ductile of the UFG Cu/Ni clad foil. The strain rate sensitivity of the UFG Ni layer is significantly higher than that of the UFG Cu layer, which leads to a gradual increase in the stress gradient with increasing strain rate. Large stress gradients promote the storage of dislocations and indirectly inhibit the recovery of dislocations. However, this does not apply to CG Cu/Ni clad foils due to the low strain rate sensitivity of CG Cu and Ni, and the increase in strain rate does not enhance the stress gradient near the interface layer. In terms of strain hardening, the increasing elongation is associated with the length of the fourth stage in Figure 6. The third stage of work hardening is related to the reversion of the cross-slip of the screw dislocations, and the fourth stage is associated with the climbing of the edge dislocations [36]. In Figure 10b,c, the interface mismatch forms many edged dislocations due to the different lattice constants. Further, few screw dislocations appear after annealing. In the third stage, fewer screw dislocations at the interface layer recover quickly under the cross-slip recovery effect. Thus, the third stage contains a small strain range, and the cross-slip reversion of screw dislocations clears the dislocation plugging barrier under high stress. The intrinsic mechanism of the fourth stage is that the density of screw dislocations stops increasing and the density of edged dislocations still grows until the necking is caused by the increased climbing effect of edged dislocations (*θ* = 0) [37,38]. For Cu/Ni clad foils, under high strain rate conditions, a large number of mobile edged dislocations generate dislocation plugging at the interface, and the mismatched interface hinders the movement of edged dislocations. The climbing behavior of edged dislocations is suppressed to increase the length of the fourth stage in Figure 6. Thus, the ductility of the Cu/Ni clad foil is improved. This performance is reflected in the fracture morphology in Figure 9, and the rough fracture morphology and entangled slip bands with different orientations appear near the interface layer at $\dot{\varepsilon }=10^{-1}{\;s}^{-1}$. As $\dot{\varepsilon }=10^{-4}{\;s}^{-1}$, the fracture surface is relatively smooth and the slip lines are regular and isotropic, reflecting that the dislocations undergo sufficient reversion at low strain rates (cross-slip of screw dislocations and climbing of edge dislocations). This condition effectively alleviates dislocation plugging and reduces strain hardening so that the ductility of Cu/Ni clad foils at low strain rates is lower than at high strain rates.

## 5. Conclusions

The present study was designed to investigate the strain rate sensitivity of CG Cu/Ni clad foils in the quasi-static strain rate range ($\dot{\varepsilon }=10^{-4}{\;s}^{-1}\sim 10^{-1}{\;s}^{-1}$). The results showed that CG Cu/Ni clad foils exhibited higher strain rate sensitivity and lower activation volume than CG pure Cu and Ni. The strain rate sensitivity *m* value was in the range of 0.012~0.015, which is three times the value of *m* for CG pure Cu. Strength and ductility increased with strain rate. The elongation was raised about 10% for specimens with different grain sizes by increasing the strain rate from $10^{-4}\;s^{-1}$ to $10^{-1}{\;s}^{-1}$. The rough fracture morphology and entangled slip bands with different orientations appeared at a higher strain rate near the interface layer. The interfacial layer with high dislocation density reduced the activation and improved the strain hardening sensitivity. At high strain rates, many edge dislocations were activated and generated severe interactions to suppress the climbing effect. It extended the fourth stage of strain hardening to improve ductility. Furthermore, the mechanical properties of Cu/Ni clad foils were still influenced by size effects in the quasi-static load strain rate range. Further research might explore the performance of Cu/Ni clad foils at ultra-high strain rates.

## Data Availability

The raw/processed data required to reproduce these findings cannot be shared at this time as the data also forms part of an ongoing study.
